# Vestibular Deficits Following Concussion

**DOI:** 10.15844/pedneurbriefs-29-5-2

**Published:** 2015-05

**Authors:** J. Gordon Millichap

**Affiliations:** 1 Division of Neurology, Ann & Robert H. Lurie Children's Hospital of Chicago, Chicago, IL; 2 Departments of Pediatrics and Neurology, Northwestern University Feinberg School of Medicine, Chicago, IL

**Keywords:** Pediatric, Concussion, Vestibular Deficits, Cognitive Testing

## Abstract

Investigators from the Division of Emergency Medicine, Sports Medicine, and Department of Pediatrics, Children's Hospital of Philadelphia, PA, and Sports Medicine, Somerset, NJ, performed a retrospective cohort study of 247 patients ages 5-18 years with concussion referred from July 2010 to Dec 2011; 81% of patients showed a vestibular abnormality on initial clinical examination.

Investigators from the Division of Emergency Medicine, Sports Medicine, and Department of Pediatrics, Children's Hospital of Philadelphia, PA, and Sports Medicine, Somerset, NJ, performed a retrospective cohort study of 247 patients ages 5-18 years with concussion referred from July 2010 to Dec 2011; 81% of patients showed a vestibular abnormality on initial clinical examination. The Vestibular/Ocular Motor screening assessment includes tests for dysmetria, nystagmus, smooth pursuits, fast saccades, and gaze stability, near-point convergence, and gait/balance testing. Vestibular deficits included either abnormal vestibular ocular reflex (VOR) or abnormal tandem gait testing. Patients with vestibular clinical signs initially took a significantly longer time to return to school (median 59 days vs 6 days, P=.001) or to be fully cleared (median 106 days vs 29 days, P=.001). They scored more poorly on initial neurocognitive testing and they took longer to recover from neurocognitive deficits. Patients with 3 or more previous concussions had a greater prevalence of vestibular deficits, and it took longer for those deficits to resolve. Vestibular rehabilitation therapy is recommended. [1]

COMMENTARY. While vestibular deficits as symptoms of concussion are well recognized [2], the correlation of persistent signs of vestibular dysfunction with extended recovery times and neurocognitive deficits are not previously reported. Vestibular dysfunction is a prevalent sequel to sports and recreation-related concussions in children and adolescents, and vestibular rehabilitation is important in their management. Since recovery is often slow and prolonged, causes of vertigo and ataxia other than trauma may need to be considered in diagnosis and treatment. These include infection (otomastoiditis, labyrinthitis, vestibular neuritis, herpes zoster oticus), vertebrobasilar insufficiency, and benign paroxysmal positional vertigo. The injury to the vestibular system may be central (vestibular nuclei and cerebellum) or peripheral (semicircular canals, otoliths, vestibular nerve). The location of the injury may lead to a specific form of therapy.

Vestibular paroxysmia is a rare but treatable cause of short vertigo attacks related to arterial compression of the eighth cranial nerve. Usually found in adults, a report of three pediatric cases, ages 8, 9, and 12 years, is noteworthy. Brief, vertiginous attacks with nystagmus occurred several times a day. MRI revealed arterial compression of the vestibular nerve. Attacks were controlled by low-dose carbamazepine (2-4 mg/kg daily) [3].
